# Persistent Müllerian Duct Syndrome: Understanding the Challenges

**DOI:** 10.1155/2022/2643833

**Published:** 2022-03-27

**Authors:** Irene Chua, Naeem Samnakay

**Affiliations:** ^1^Department of Paediatric Surgery, Perth Children's Hospital, Western Australia, Australia; ^2^Division of Surgery, Medical School, The University of Western Australia, Western Australia, Australia

## Abstract

Persistent Müllerian duct syndrome (PMDS) is a rare autosomal recessive condition defined by the presence of Müllerian duct-derived structures in an otherwise normally masculinized phenotypical and genotypical (46,XY) male. We describe the case of an infant diagnosed with PMDS, managed and followed up for 7 years. The diagnosis of PMDS was made at laparoscopy at 6 months of age for investigation and management of bilateral impalpable testes. A Müllerian structure resembling a uterus with bilateral fallopian tube-like structures was seen in the pelvis, along with bilateral intra-abdominal testes. Gonadal biopsy confirmed normal testicular tissue. The child underwent successful bilateral two-stage Fowler-Stephens orchidopexies. The Müllerian remnant was preserved to maintain testicular vascularity. At the most recent follow-up, the testes are intrascrotal and normal on palpation. There have been no clinical symptoms or concerns with the Müllerian remnant during surveillance with ultrasound and MRI. To date, there are less than 300 cases described in the medical literature, with limited consensus on management. We reflect on challenges the condition poses, including fertility preservation in PMDS, testicular and Müllerian malignancy risk in PMDS, and optimal management and surveillance of PMDS.

## 1. Introduction

Persistent Müllerian duct syndrome (PMDS) is a rare genetic condition defined by the presence of Müllerian duct-derived structures (fallopian tubes, uterus, and upper vagina) in an otherwise normally masculinized phenotypical and genotypical (46,XY) male. Accurate incidence remains largely unknown. There are less than 300 cases described in the medical literature, with various management options for the condition described [1–3]. In this paper, we present a case of PMDS diagnosed and managed in infancy, with follow-up over a 7-year period. We review the current up-to-date evidence for the management of PMDS and reflect on challenges the condition poses.

## 2. Case Presentation

A healthy, first-born 6-month-old male infant was referred by a pediatrician for the management of bilateral impalpable testes. The infant was born at term, with no significant family history or consanguinity between parents. On examination, he was noted to have a normal penis with complete prepuce and glanular urethral meatus. The scrotum was well formed but empty. The testes were impalpable bilaterally. Initial ultrasound scan of the scrotum and pelvis did not identify the testes and did not detect any pelvic abnormality.

**Figure 1:**
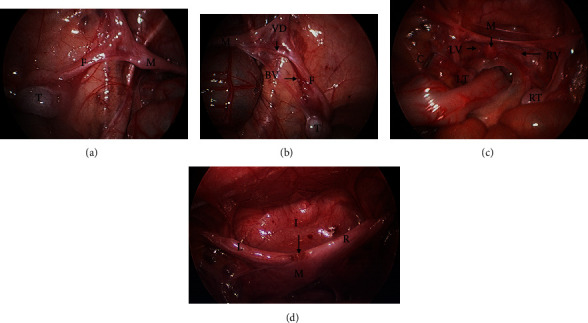
(a) Diagnostic laparoscopy showing the left testicle and fallopian tube-like structure. Key: M: persistent Müllerian duct structure; F: fallopian tube-like structure; T: left testicle. (b) The right testicle and fallopian tube-like structure. Key: M: persistent Müllerian duct structure; F: fallopian tube-like structure; T: right testicle; VD: right vas deferens closely associated with fallopian tube-like structure; BV: blood vessels along vas and fallopian tube-like structure. (c) Laparoscopic view showing completed first-stage FSO bilaterally. Key: M: persistent Müllerian duct structure; RV: right vas deferens; RT: right testis; LV: left vas deferens; LT: left testis; C: clips on the distal end of left gonadal vessels. (d) Laparoscopic view illustrating completed second-stage bilateral FSO. Small midline incision at the dome of persistent Müllerian duct structure was made to allow tension-free orchidopexy. Key: M: persistent Müllerian duct structure; I: midline incision in the Müllerian duct structure; R: right fallopian tube-like structure with associated vas and blood vessels exiting the abdominal wall; L: left fallopian tube-like structure with associated vas and blood vessels exiting the abdominal wall.

Diagnostic laparoscopy for impalpable testes at age 6 months revealed bilateral intra-abdominal testes, with normal and expected testicular structure and appearance. However, a Müllerian structure resembling the uterus, with bilateral fallopian tubes and round ligaments, was also noted. Vasa deferentia were clearly identified in close association with the fallopian-tube like structures (Figures 1(a) and 1(b)). A pole-to-pole gonadal biopsy was performed to assess gonadal histological structure. Cystourethroscopy did not reveal an obvious channel between the Müllerian structure and the urethra. Although PMDS was clinically suspected, no further surgery was performed at this stage. Parents were counselled about the operative findings; results of gonadal biopsy, endocrine work-up, and karyotype were awaited.

Histology of bilateral gonadal biopsy showed normal testicular tissue. Sex-determining region (SRY) on the Y chromosome was present on fluorescence in situ hybridisation. 46,XY karyotype was confirmed. Hormonal studies revealed age-appropriate levels of luteinising hormone (LH), follicle stimulating hormone (FSH), and testosterone. Serum anti-Mullerian hormone (AMH) was normal for age (serum AMH level 526 pmol/L; normal range for <1 year old: 454-944 pmol/L). HCG stimulation test showed robust stimulation of testosterone production (baseline level was <1 nmol/L, and stimulated level 72 hours later was 18 nmol/L).

The child underwent a planned bilateral laparoscopic two-stage Fowler-Stephens orchidopexy (FSO). In order for sufficient mobility for the testes to be brought down, the limiting length of the gonadal vessels was divided (Figure 1(c)). The vasa and associated vasal vasculature ran in close association with the fallopian tube-like arms of the Müllerian structure (Figure 1(b)). The Müllerian structure was therefore left intact to limit any damage to the vas or vessels. A small midline longitudinal apical incision was made in the Mullerian structure to allow greater splay of the fallopian tube-like arms and facilitate tension free orchidopexy (Figure 1(d)). Both intra-abdominal testes were successfully moved to an intrascrotal position with the vasa and vasal blood supply intact.

**Figure 2:**
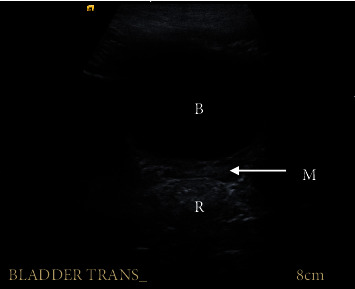
Müllerian remnant viewed on surveillance pelvic ultrasound scan. Key: M: Müllerian remnant; B: bladder; R: rectum.

**Figure 3:**
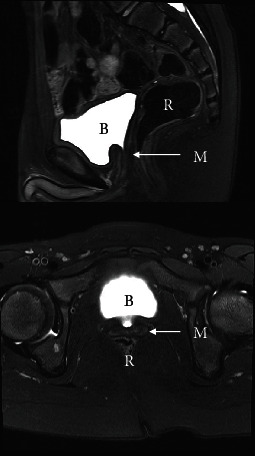
Magnetic resonance imaging (MRI) sagittal and axial views showing Müllerian remnant. Key: M: Müllerian remnant; B: bladder; R: rectum.

The child has had annual follow-up with pelvic and scrotal surveillance ultrasound for seven years, plus a pelvic baseline MRI at age six. The Müllerian structure has remained small and stable on ultrasound follow-up (Figure 2). The testes are both intrascrotal and normal in structure and volume for age. On MRI of the pelvis, the Müllerian remnant was well visualised and small (Figure 3). Further periodic surveillance, MRI would be a useful adjunct to annual USS surveillance. The child has not had any urological symptoms relating to the Müllerian structure, such as epididymitis, urinary tract infection, or urinary incontinence.

## 3. Discussion

First described in the literature in 1939 by Nilson [4], PMDS is a rare disorder of internal male sex development, with persistence of Müllerian duct-derived structures in addition to Wolffian duct-derived structures. PMDS is transmitted as an autosomal recessive condition [3]. It may also occur as a sporadic *de novo* mutation.

Sexual dimorphism, although determined genetically at the time of fertilisation, does not occur until the seventh week of embryological development. Initially, the embryo possesses two pairs of genetic ducts, the Wolffian and Müllerian ducts. The presence of SRY on the Y chromosome leads to differentiation of the primordial gonad into testis. The process of male sex development depends on testosterone, dihydrotestosterone, and anti-Müllerian hormone (AMH) [5]. At the end of the seventh gestational week, AMH is produced by fetal Sertoli cells. This leads to the regression of the Müllerian duct and its derivatives. Failure to do so leads the Müllerian duct to differentiate into uterus, fallopian tubes, and upper vagina. In addition, the presence of Leydig cells results in the secretion of testosterone, which directs localised differentiation of Wolffian duct structures including the epididymis, vas deferens, seminal vesicles, and ejaculatory ducts.

It is a defect in AMH or AMH receptors which results in the manifestation of PMDS. It is estimated that 85% of cases of PMDS occur because of a mutation in either the AMH synthesis gene on chromosome 19 (type I PMDS, 45%) or a defect in the AMH receptor (AMHR) gene on chromosome 12 (type II PMDS, 40%) [6, 7]. In the remaining, the cause for PMDS is unknown (idiopathic PMDS). In our described case, serum AMH levels were normal, suggesting a likely AMHR mutation as the underlying aetiology. Genetic testing to clarify the likely molecular aetiology has not been performed as yet but is an important consideration.

The defect results in the coexistence of both Wolffian and Müllerian duct structures in a phenotypic male, as external virilisation is complete due to the presence of testosterone.

PMDS usually presents incidentally in a male presenting with cryptorchid testes or inguinal hernia in childhood or sometimes in adulthood or testicular tumor or abdominal mass in adulthood [3, 8, 9].

**Figure 4:**
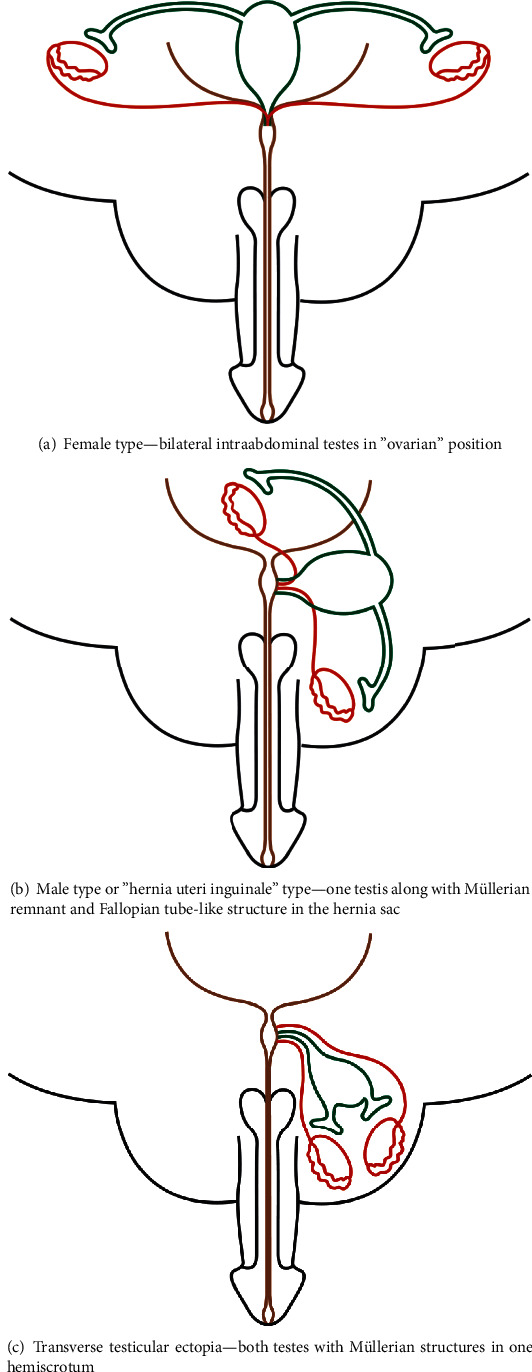
Three described anatomical variants of PMDS. Key: red: Wolffian structures plus testes; green: persistent Müllerian structures; brown: bladder and urethra.

PMDS is classified based on the location of testes and Müllerian structures. There are three clinical anatomical variants described in the literature (Figure 4).

*PMDS-female type (FT)*. This is the most common type reported in the literature (Figure 4(a)). In FT PMDS, both testes are intra-abdominal, in the ovarian position, with the vasa and associated vasculature closely applied to the fallopian tube-like structures. Gonadal biopsy usually demonstrates normal testicular structure on histology. Our described case had FT PMDS.

In children, preservation of fertility potential and hormonal function should be an overriding aim. Gonadal malignancy risk reduction should be maximised by early orchidopexy, as for any child with intra-abdominal testes.

*PMDS-hernia uteri inguinale (HUI)*. This is the next most commonly reported variant (Figure 4(b)). It is also known as male-type PMDS. HUI usually presents with an ipsilateral hernia with ipsilateral descended testis and Müllerian structures within the hernia. The contralateral testis is usually undescended or intraabdominal.

*PMDS-transverse testicular ectopia (TTE)*. This is considered the rarest type of PMDS (Figure 4(c)). Both testes and the Müllerian structures are herniated into one hemiscrotum [10, 11]. Not all cases of TTE are associated with PMDS. Type 1 TTE comprises up to 50% of cases, associated with inguinal hernia only. Type 2 TTE comprises up to 30% cases, associated with persistent Müllerian structures. Type 3 TTE is associated with other anomalies such as scrotal anomalies, hypospadias, fused vas deferens, and horseshoe kidney [12].

## 4. Clinical Management of PMDS in Children: Evidence and Challenges

The surgical management and subsequent follow-up of PMDS may not have conformity, but some important guiding principles are highlighted by reviewing the literature.

### 4.1. Principle 1: Recognition and Diagnosis of PMDS

With investigative laparoscopy for impalpable testes now commonplace, the recognition of FT PMDS in infancy has increased [3]. When both Müllerian and Wolffian structures are present together at laparoscopy, it is generally considered prudent to make a careful assessment for other possible variants or differences of sex development, such as gonadal dysgenesis. Mixed gonadal dysgenesis is unlikely in the presence of a normally formed penis. If there is any doubt, it is recommended to initially perform gonadal biopsies to confirm that they comprise testicular tissue. Gonadal biopsy and karyotype before proceeding to orchidopexy will help to give the family time to understand the condition, to get reassurance about the karyotype, and to get objective reassurance about the presence of normal testicular tissue.

HUI and TTE both commonly present with inguinal hernia. During hernia repair, the Müllerian structures are noted. In some cases, the Müllerian structures are left in situ to preserve testicular vascularity and ductal structures [13]; in other reports, the Mullerian structures are excised, especially in adults [12, 14]. In children with HUI, it is recommended that the cryptorchid contralateral testis undergo orchidopexy. In adults presenting with HUI, the undescended or intraabdominal testis is often excised due to concerns about malignancy risk.

In children with TTE, the testes can be placed in separate hemiscrota by transseptal orchidopexy [15].

### 4.2. Principle 2: Preserving Gonadal Function and Fertility Potential

The literature documents fertility impairment in adults with PMDS [3]; however, there are reports of fertility and paternity in men with PMDS, including in FT PMDS [3, 16–18].

It is not clear what contributes, and to what degree, to the reported fertility impairment—it may be due to uncorrected cryptorchidism, or due to coexisting congenital gonadal and Wolffian duct anomalies, or due to ischemic and structural damage to the vas and testis secondary to excision of Müllerian remnants.

In children with PMDS, the gonads show normal testicular tissue on biopsy but are undescended [2]. The benefit of early orchidopexy in potentiating fertility is well established in the literature for cryptorchidism in general [19]. Thus, for children with PMDS, early orchidopexy to place the testes into the scrotum is indicated. Care should be taken to maximise preservation of gonadal vascularity and limit any damage to Wolffian ductal structures during hernia repair, orchidopexy, and, if deemed necessary, excision of Müllerian structures. Priority should be given to gonadal preservation and successful orchidopexy. For example, in FT PMDS, if excision of the Müllerian remnant is considered likely to damage the vasal vessels supplying the testes or the vas and ductal structures, then it should preferably be left in situ and surveilled [13]. If despite sacrificing gonadal vessels, the descent of the testes is limited by the short length of the fallopian tubes, the literature describes dividing the main body of the Mullerian structure sagittally to allow lateral splitting of each half, thus giving extra length for testicular descent without compromising the vasa or testicular vasculature (Figure 1(d)) [20].

### 4.3. Principle 3: Reducing Future Gonadal Malignancy Risk

The reported incidence of malignant change in the testes in PMDS generally ranges from 5 to 18%, a rate which is similar to abdominal undescended testes in men without PMDS. However, some reviews suggest gonadal malignancy risk in PMDS is as high as 33% [3]. Seminomas are most commonly recorded, but other Germ cell neoplasia in situ- (GCNIS-) derived testicular tumours are also reported [3].

Orchidopexy early in childhood has been shown to reduce malignancy risk in cryptorchidism [19]. Early orchidopexy should be offered to children with PMDS.

### 4.4. Principle 4: Being Aware of the Risk of Malignancy in Müllerian Remnants

The rate of Müllerian malignancy in the literature ranges from 3.1% to 8.4% [3]. In one paper describing malignancies in Müllerian remnants, 3 of the 11 described malignancy cases qualified as having PMDS [1]. Described Müllerian malignancies in PMDS include adenocarcinomas and adenosarcoma [8].

The risk of Müllerian malignancy is generally considered lower than the risk of malignancy in the associated undescended testes. The age of documented Müllerian malignancy in PMDS ranges from as young as 4 years old to 68 years old [1]. How to manage this risk of malignancy remains unclear.

If the Müllerian remnant is left in situ, the literature advocates regular ultrasound surveillance, such as annually, to assess for change in size or new mass lesions [21]. Soft tissue MRI is also a useful modality to follow up with and assess for changes in the Müllerian remnant. At present, there is no data to suggest whether one modality is superior for surveillance purposes, and there is no data to guide the frequency of surveillance should it be undertaken. If a change is detected on surveillance, then appropriate intervention will need to be undertaken.

## 5. Conclusion

PMDS is a rare disorder of internal male sexual development. The main surgical considerations are repairing associated inguinal hernias; preserving gonadal function and fertility and minimising the risk of malignant change by early orchidopexy and careful preservation of gonadal vasculature and vasal and ductal structures; and long-term surveillance for potential malignancy in pexed testes and retained Müllerian structures.

## Data Availability

Patient medical records are to be kept private and deidentified as per request of family who have consented kindly to this case report.
